# Dihydroartemisinin Regulated the MMP-Mediated Cellular Microenvironment to Alleviate Rheumatoid Arthritis

**DOI:** 10.34133/research.0459

**Published:** 2024-09-10

**Authors:** Qiuyan Guo, Qixin Wang, Jiayun Chen, Minghong Zhao, Tianming Lu, Zuchang Guo, Chen Wang, Yin Kwan Wong, Xueling He, Lin Chen, Wenjing Zhang, Chuanhao Dai, Shengnan Shen, Huanhuan Pang, Fei Xia, Chong Qiu, Daoyuan Xie, Jigang Wang

**Affiliations:** ^1^ State Key Laboratory for Quality Ensurance and Sustainable Use of Dao-di Herbs, Artemisinin Research Center, and Institute of Chinese Materia Medica, China Academy of Chinese Medical Sciences, Beijing 100700, China.; ^2^ Department of Physiology, Yong Loo Lin School of Medicine, National University of Singapore, Singapore, Singapore.; ^3^ Chinese Medical Association, Beijing 100710, China.; ^4^ Laboratory of Translational Medicine Research, Deyang People’s Hospital of Chengdu University of Traditional Chinese Medicine, Deyang 618000, China.; ^5^ State Key Laboratory of Antiviral Drugs, School of Pharmacy, Henan University, Kaifeng 475004, China.; ^6^ Department of Critical Care Medicine, Guangdong Provincial Clinical Research Center for Geriatrics, Shenzhen Clinical Research Centre for Geriatrics, Shenzhen People’s Hospital (The Second Clinical Medical College, Jinan University; The First Affiliated Hospital, Southern University of Science and Technology), Shenzhen 518020, Guangdong, China.

## Abstract

Rheumatoid arthritis (RA) is an autoimmune disease with features of synovial inflammation, cartilage erosion, bone destruction, and pain and is currently lacking a satisfactory treatment strategy. Dihydroartemisinin (DHA), the active metabolite of artemisinin, has exhibited outstanding suppressive effects on RA without obvious side effects. However, the underlying mechanisms remain unclear, which limits its further clinical application. The purpose of this study is to reveal the pharmacodynamic mechanism of DHA against RA by means of a combination of single-cell RNA sequencing (RNA-seq), proteomics, as well as transcriptomics both in vivo and in vitro. In our results, DHA effectively reduced the degree of redness, swelling, and pain in RA rats and dramatically changed the synovial tissue microenvironment under the pathological state. Within this microenvironment, fibroblasts, macrophages, B cells, and endothelial cells were the major affected cell types, primarily through DHA targeting the extracellular matrix (ECM) structural constituent signaling pathway. In addition, we confirmed that DHA regulated the ECM by modulating matrix metalloproteinase 2 (MMP2) and MMP3 in the synovial tissue of RA rats. Moreover, DHA induced apoptosis in MH7A cells, further validating the bioinformatics data. In conclusion, DHA effectively reduced the inflammatory response and improved the immune microenvironment in synovial tissue by inhibiting MMP2 and MMP3. Our findings provide a basis for the application of DHA in the treatment of RA.

## Introduction

Rheumatoid arthritis (RA) is a prevalent autoimmune disease that results in persistent inflammation of the synovium in limb joints, leading to progressive bone destruction [1]. Within a span of 10 years, the disability rate can reach as high as 60% [2]. While the definite cause of RA remains unclear, it is widely acknowledged that synovial lesions are closely associated with its development. Presently, the primary clinical approaches for RA control involve nonsteroidal anti-inflammatory drugs, antirheumatic drugs, glucocorticoids (GCs), and other nonspecific medications [3,4]. However, methotrexate (MTX) and GCs, as first-line treatments, only provide partial relief from arthritis symptoms for some RA patients, and they may not be suitable for all individuals [5]. Additionally, these medications can sometimes lead to hepatorenal toxicity, gastrointestinal issues, and other adverse reactions [6–8]. Therefore, the development of targeted innovative drugs holds great significance.

Dihydroartemisinin (DHA), the active metabolite of artemisinin, offers advantages such as enhanced water solubility, reduced toxicity, and fewer side effects compared to artemisinin. DHA attracts the attention of researchers due to its potential in treating various immune inflammatory conditions, including RA [9–13]. As reported, DHA effectively reduces ankle joint swelling in rats with collagen-induced arthritis [14]. Fan et al.’s [15,16] research indicates that DC32, a DHA derivative, induces p62 expression and effectively inhibits RA by up-regulating Nrf2 expression at the gene and protein levels. While DHA is considered a promising drug for improving RA, further research is needed to identify specific targets and molecular mechanisms.

RNA sequencing (RNA-seq) is a powerful method for analyzing changes in gene expression at the individual cell level. It enables a comprehensive understanding of the cellular responses to drug actions and provides a novel approach to studying the mechanisms underlying drug effects [17]. Recent studies utilizing single-cell RNA-seq (scRNA-seq) analysis on inflamed joint tissues have shed light on the intricate nature of synovitis, a critical pathological feature contributing to the development of RA. In previous studies that applied scRNA-seq to investigated RA synovitis, fibroblasts [18], macrophages [19] B cells [20,21], and T cells [22,23] are the major cell types closely associated with synovitis. Fibroblasts and macrophages, as the primary components of the synovium, play pivotal roles in articular cartilage and bone destruction, making them important targets for RA treatment. The scRNA-seq technology has already been employed in the investigation of synovial tissue [24]. To sum up, by utilizing scRNA-seq technology, we can gain deeper insights into the molecular mechanisms underlying how drugs work during the promotion of the RA joint synovial repair. Thus, we applied scRNA-seq in exploring the intrinsic mechanism of DHA improving RA.

To provide scientific evidence for the clinical use of DHA, the purpose of this study is to reveal the immunoregulatory mechanisms through which DHA intervenes in rheumatoid synovitis and associated pain at the cellular, protein, and gene levels. First, we established a classic adjuvant-induced RA rat model to evaluate the characteristics of DHA. Subsequently, we analyzed the impact of DHA on the synovial tissue microenvironment at the single-cell and protein levels using single-cell sequencing and proteomics, respectively. Furthermore, we investigated the molecular mechanisms by which DHA alleviates pain at the gene level in the hippocampus.

## Results

### DHA effectively improved the symptoms of RA rats

**Fig. 1.**
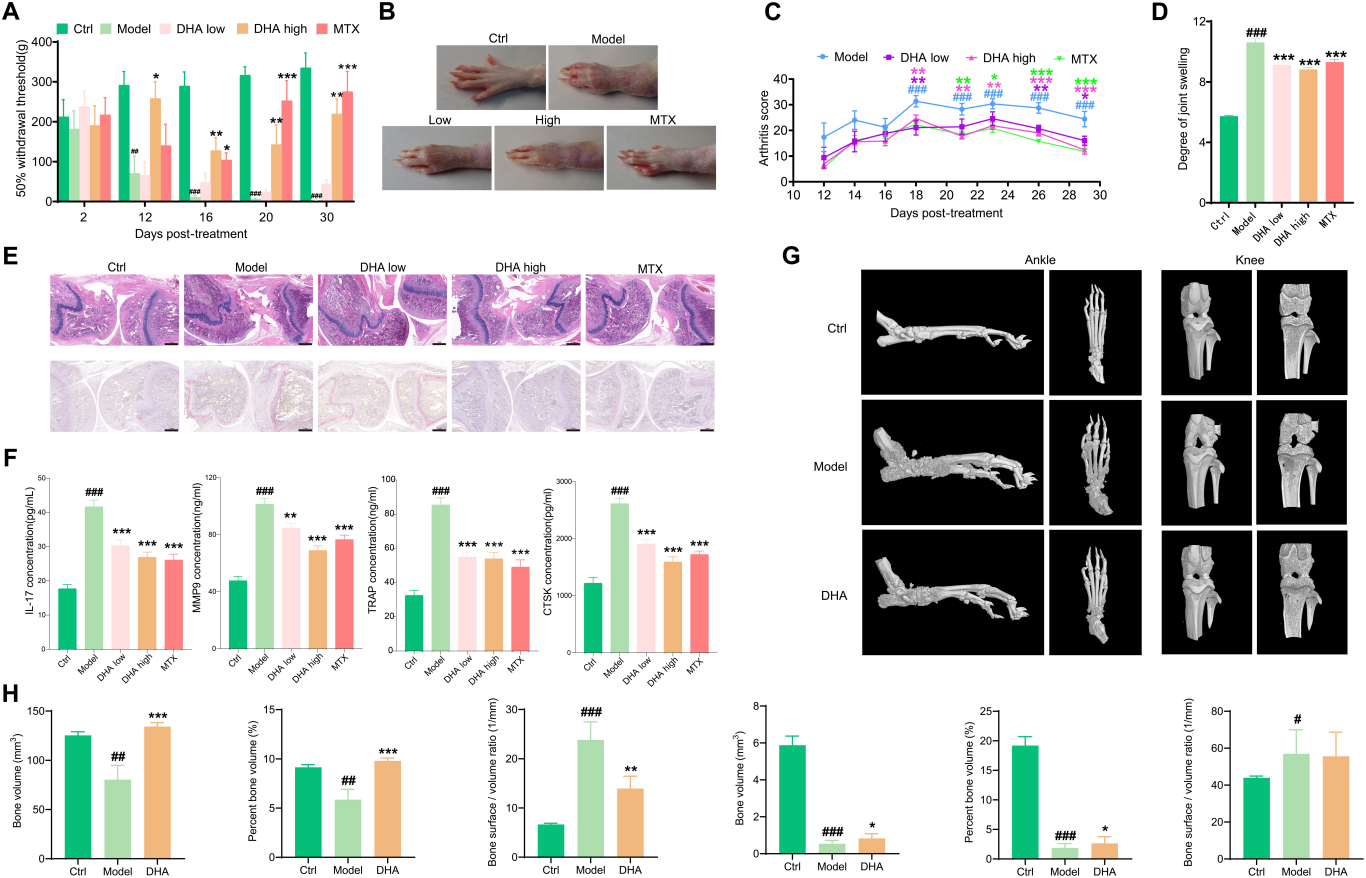
Effect of DHA on RA rats. (A) Mechanical pain threshold of rats during the DHA administration. (B) Left hind limb ankle photos of rats after the DHA administration. (C) Arthritis index of rats during the DHA administration cycle. (D) Joint swelling of rats at the end of administration. (E) H&E and TRAP staining of knee joints (40×). (F) Effects of DHA on IL-17, MMP9, TRAP, and CTSK in rats’ serum (*n* = 6). (G) Morphological changes of ankle and knee joints of AIA rats. (H) Impact of DHA on bone microstructure parameters of the ankle (left 3 panels) and knee joints (right 3 panels) (*n* = 3). The data are presented as the mean ± SD. ^#^*P* < 0.05, ^##^*P* < 0.01, ^###^*P* < 0.001 compared to the Control (Ctrl) group respectively; *P < 0.05, **P < 0.01, ***P < 0.001 compared to the Model group respectively.

The von Frey nociceptive assay is used to assess pain sensitivity in RA rats. Arthritis scoring was performed by observing the pain manifestation, swelling, and mobility of the ankle and knee joints of rats to visualize the pharmacodynamic effects of DHA. In this study, we observed that DHA improved the reduction in mechanical pain threshold (Fig. 1A) and resulted in varying degrees of reduction in redness and swelling of the fingers and ankles in the adjuvant induced arthritis (AIA) model rats (Fig. 1B). Throughout the administration period, DHA effectively reduced arthritis scores and joint swelling (Fig. 1C and D). Additionally, aberrant expression of IL-17, matrix metalloproteinase 9 (MMP9), tartrate-resistant acid phosphatase (TRAP), and cathepsin K (CTSK) was markedly and positively correlated in patients with early RA, and these indicators are important in assessing the extent of RA disease. DHA effectively inhibited the cartilage and joint destruction caused by inflammatory invasion and the secretion of cytokines interleukin-17 (IL-17), MMP9, TRAP, and CTSK (Fig. 1E and F). Furthermore, bone morphology of rat knee and ankle joints was analyzed by x-ray and micro-CT (computed tomography) to visualize the effects of DHA on the bones and joints of RA rats. DHA notably prevented bone loss in both the ankle and knee joints of RA rats (Fig. 1G and H).

### DHA altered the cellular heterogeneity of synovial tissue under RA conditions

**Fig. 2.**
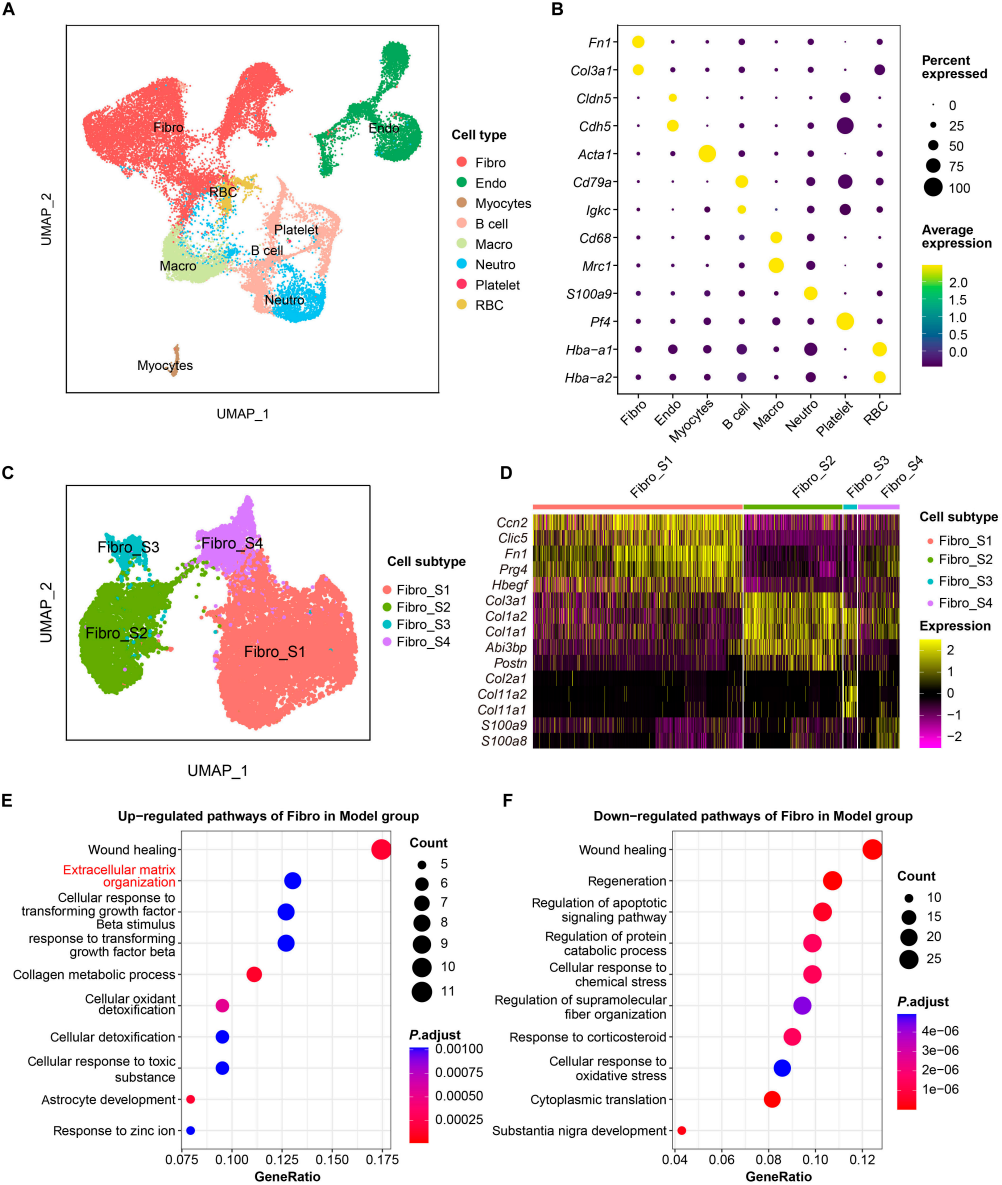
The treatment effect of DHA upon cellular composition and fibroblast of knee synovium in RA rats. (A) The Uniform Manifold Approximation and Projection (UMAP) plot shows the presence of various cell types in the scRNA-seq dataset, including fibroblasts (Fibro), endothelial cells (Endo), myocytes, B cells, macrophages (Macro), neutrophils (Neutro), platelets, and red blood cells (RBC). (B) The bubble plot illustrates the marker genes specifically expressed by each cell type in the scRNA-seq dataset. (C) The UMAP plot shows the subclustering and annotation of fibroblasts, dividing into 4 subtypes: Fibro_S1, Fibro_S2, Fibro_S3, and Fibro_S4. (D) The heatmap shows the top 15 marker genes specifically expressed by each fibroblast subgroup. (E) The dotplot indicates the gene ontology (GO) functional pathways based on the significantly up-regulated genes of Model versus Ctrl (MVC) and Model versus DHA (MVD) groups. (F) The dotplot indicates the GO functional pathways based on the significantly down-regulated genes of MVC and MVD groups.

scRNA-seq is a robust tool for decomposing the transcriptomes of complex tissues at the single-cell level, which can be used to study the pathogenesis of RA and characterize the role of DHA. Through further single-cell profiling, we identified a total of 8 cell types in the synovial tissues according to marker genes: fibroblasts, endothelial cells, myocytes, B cells, macrophages, neutrophils, platelets, and red blood cells (Fig. 2A and B). DHA intervention induced major changes in 4 types of cells, namely, fibroblasts, macrophages, B cells, and endothelial cells.

In our study, RNA-seq analysis was employed to examine the genetic and functional changes in RA synovial fibroblast subsets treated with DHA (Fig. 2C and D). Gene ontology (GO) enrichment analysis of differentially expressed genes (DEGs) revealed that the up-regulated genes in the Model group were mainly associated with functional pathways related to extracellular matrix (ECM) composition and response to transforming growth factor-β (TGF-β) (Fig. 2E). The down-regulated genes after DHA treatment also exhibited enrichment in ECM composition (Fig. 2F). These findings confirmed that DHA can change the microenvironment of synovial tissue under RA conditions.

### DHA alleviated the conversion of macrophages from the M1 type to the M2 type and inhibited the activation of synovial membrane B cells in RA rats

**Fig. 3.**
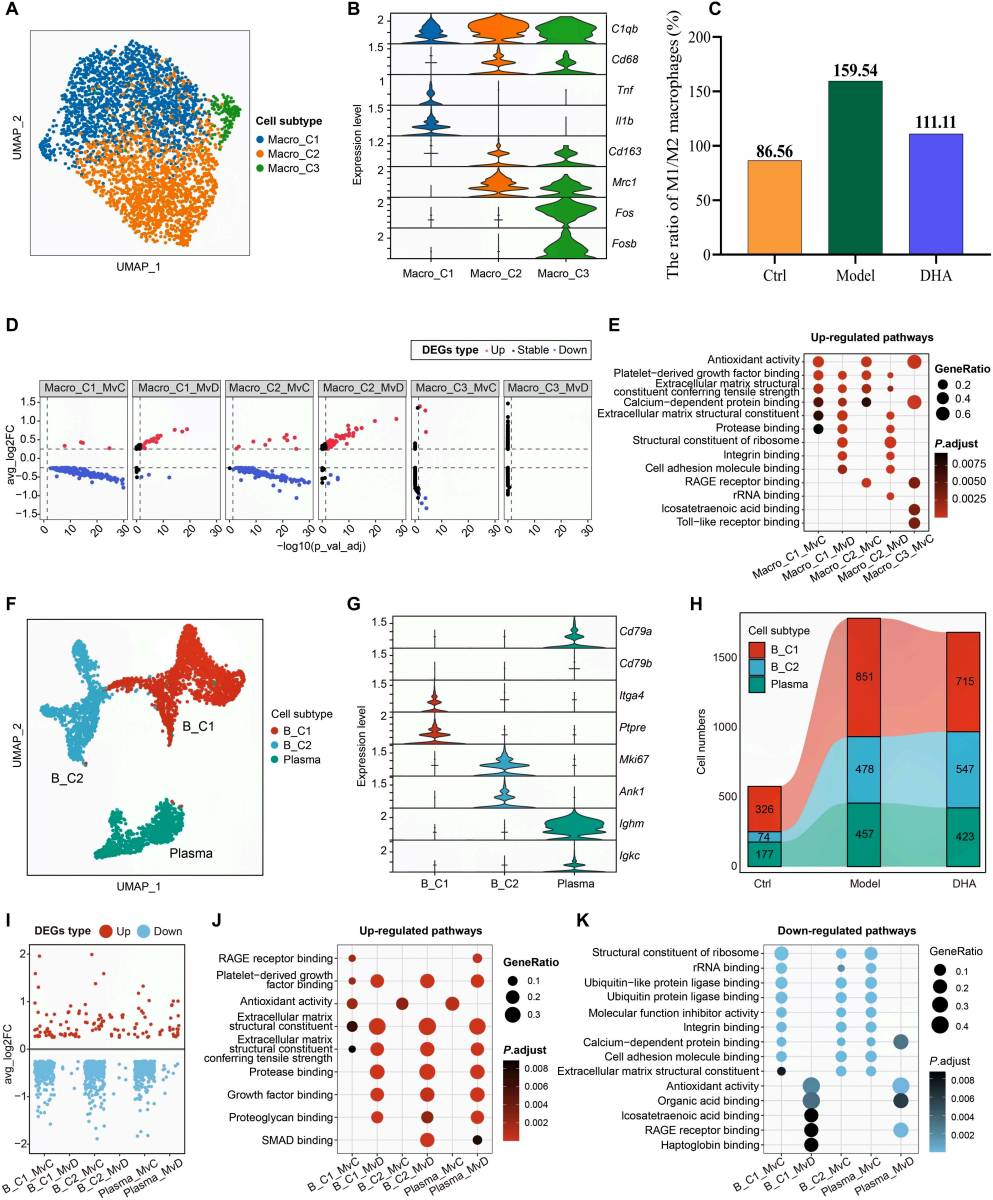
DHA exerted an anti-inflammatory effect on macrophages and B cells in RA rats. (A) UMAP plot of 3 distinct subtypes of macrophage cells. (B) The violin plot displays the relative expression levels of marker genes across different macrophage subtypes. (C) Ratio of M1/M2 macrophages in different groups. (D) The scatter dot plots illustrate the differentially expressed genes (DEGs) that were up-regulated or down-regulated in each macrophage subtype within the MVC and MVD groups. (E) GO pathway enrichment analysis was performed based on the overlapping up-regulated DEGs in all macrophage subtypes within the MVC and MVD groups. (F) The UMAP visualization demonstrates the presence of 3 distinct subtypes of B cells. (G) The violin plot displays the relative expression levels of marker genes across different B cell subtypes. (H) The Sankey diagram depicts the cell proportions and numbers of the 3 B cell subtypes in different groups. (I) The scatterplots illustrate the up-regulated or down-regulated DEGs in each B cell subtype within the MVC and MVD groups. (J and K) GO pathway enrichment analysis was performed on the overlapping up-regulated (J) and down-regulated (K) DEGs in all B cell subtypes within the MVC and MVD groups.

On the basis of different representative genes, the macrophages were categorized into 3 subtypes: Macro_C1 (M1 macrophages), Macro_C2 (M2 macrophages), and Macro_C3 (Fig. 3A and B). In contrast with normal rats, the ratio of M1/M2 macrophages increased from 86.56% to 159.54% in the Model group, whereas DHA treatment attenuated the increase to 111.11%, indicating that DHA regulated the M1/M2 ratio of macrophages in RA rats (Fig. 3C). Further GO enrichment analysis revealed the DEGs between different groups. Although among all macrophage subtypes, there were not obvious up-regulated/down-regulated DEGs of the Model versus DHA (MVD) group, as well as GO enriched pathways in the Macro_C3 subtype, the marked DEGs of Macro_C1 subtype and Macro_C2 subtype were primarily associated with the ECM structural component signaling pathway (Fig. 3D and E). These results suggested that DHA shows potential as a treatment option for RA by modulating the cellular microenvironment by enhancing the conversion of macrophages from M1 to M2 phenotype, which aligns with previous studies.

We categorized a total of 4,048 B cells into 3 subtypes: B_C1 (Itga4^+^Ptpre^+^), B_C2 (Mki67^+^Ank1^+^), and plasma (Cd79a^+^lghm^+^Igkc^+^) (Fig. 3F and G). Compared to the control group, the total number of B cells in RA rats was markedly increased, while DHA treatment could partially reverse this change (Fig. 3H). In most B_C1 subtypes, pathways associated with ECM structural components were down-regulated after DHA treatment compared to the Model group (Fig. 3I to K). These findings indicate that DHA may inhibit B cell activation by regulating the B cell immune microenvironment.

### DHA treatment inhibited the endothelial cells in RA rats

**Fig. 4.**
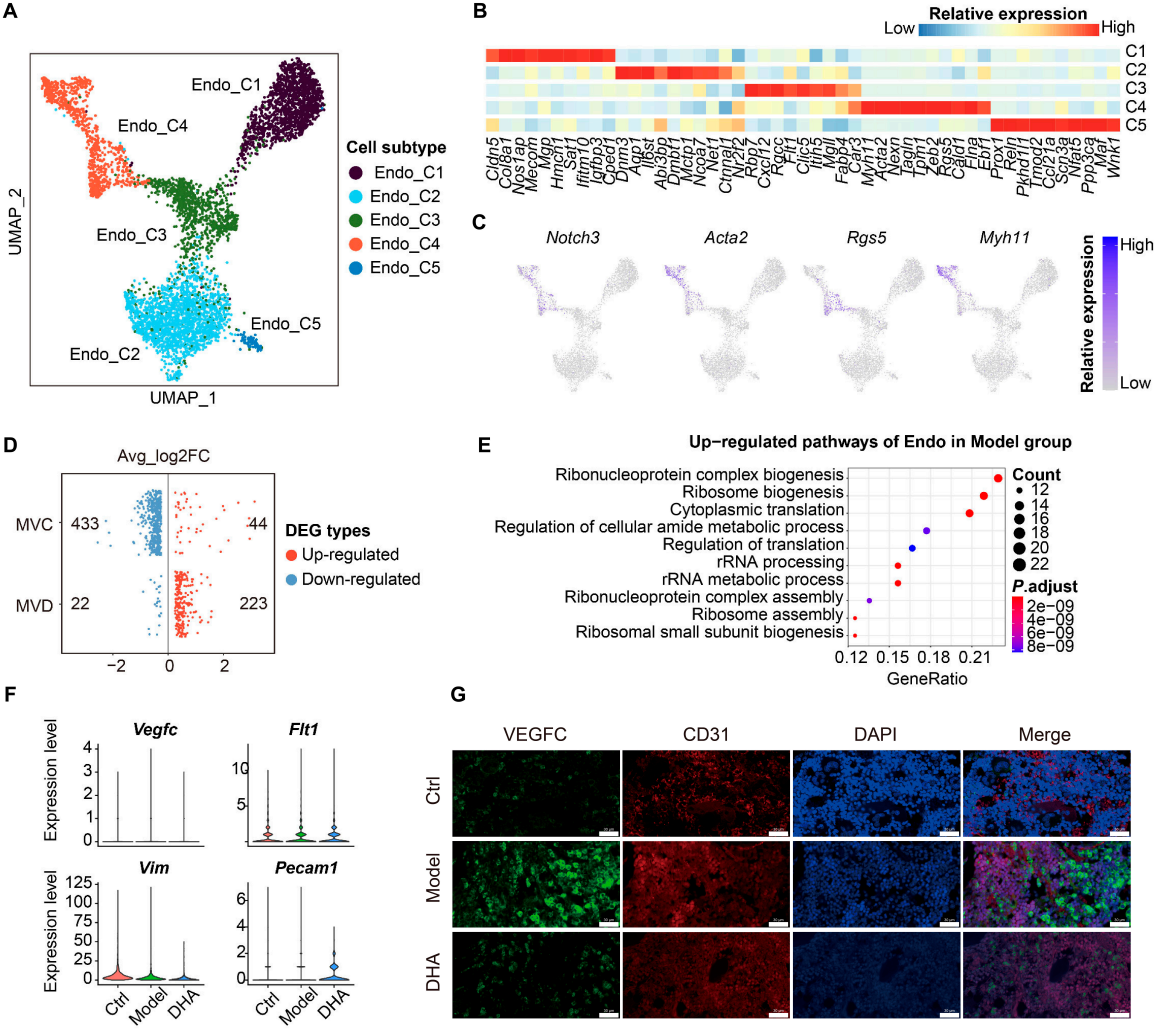
The treatment effect of DHA upon endothelial cells in rat synovial tissues. (A) The UMAP plots show the unsupervised clustering of endothelial (Endo) subtypes. (B) The heatmap plot shows the relative expression of markers of endothelial subtypes. (C) The feature plots show the relative expression of markers of endothelial subtypes. (D) The plot shows the log_2_ fold change (log2FC) value in the Model group. The scatter point in red or blue indicates the up-regulated or down-regulated DEGs, respectively. (E) The dotplot indicates the GO functional pathways based on the significantly overlapping up-regulated genes of MVC and MVD groups. (F) Violin plot shows expression of Vegfc, Flt1, Vim, and Pecam1 in the Endo across Ctrl, Model, and DHA groups. (G) Immunofluorescence images show the expression levels of VEGFC (green) and CD31 (endothelial marker, red) in rat synovial tissues.

As shown in Fig. 4A to C, we subset endothelial cells and reclustered them into 5 subtypes according to representative markers, including C1 (Mgp^+^ and Hmcn1^+^), C2 (Dnm3^+^ and Dmbt1^+^), C3 (Cxcl12^+^ and Rgcc^+^), C4 (Acta2^+^ and Tagln^+^), and C5 (Reln^+^ and Maf^+^). To clarify the transcriptomic changes of endothelial cells among the 3 (Control, Model, DHA) groups, we conducted DEG analysis (Fig. 4D). As shown, the ribosome biogenesis, regulation of translation, and ribosome assembly functions were up-regulated in the Model group (Fig. 4E). Immunofluorescence images showed decreased expression levels of vascular endothelial growth factor C (VEGFC) and CD31 (markers of endothelial cells) in rat synovial tissues, which was consistent with the single-cell transcriptome analysis results (Fig. 4F and G).

### Proteomics- and transcriptomics-based identification of key target proteins and altered hippocampal gene expression patterns by DHA

**Fig. 5.**
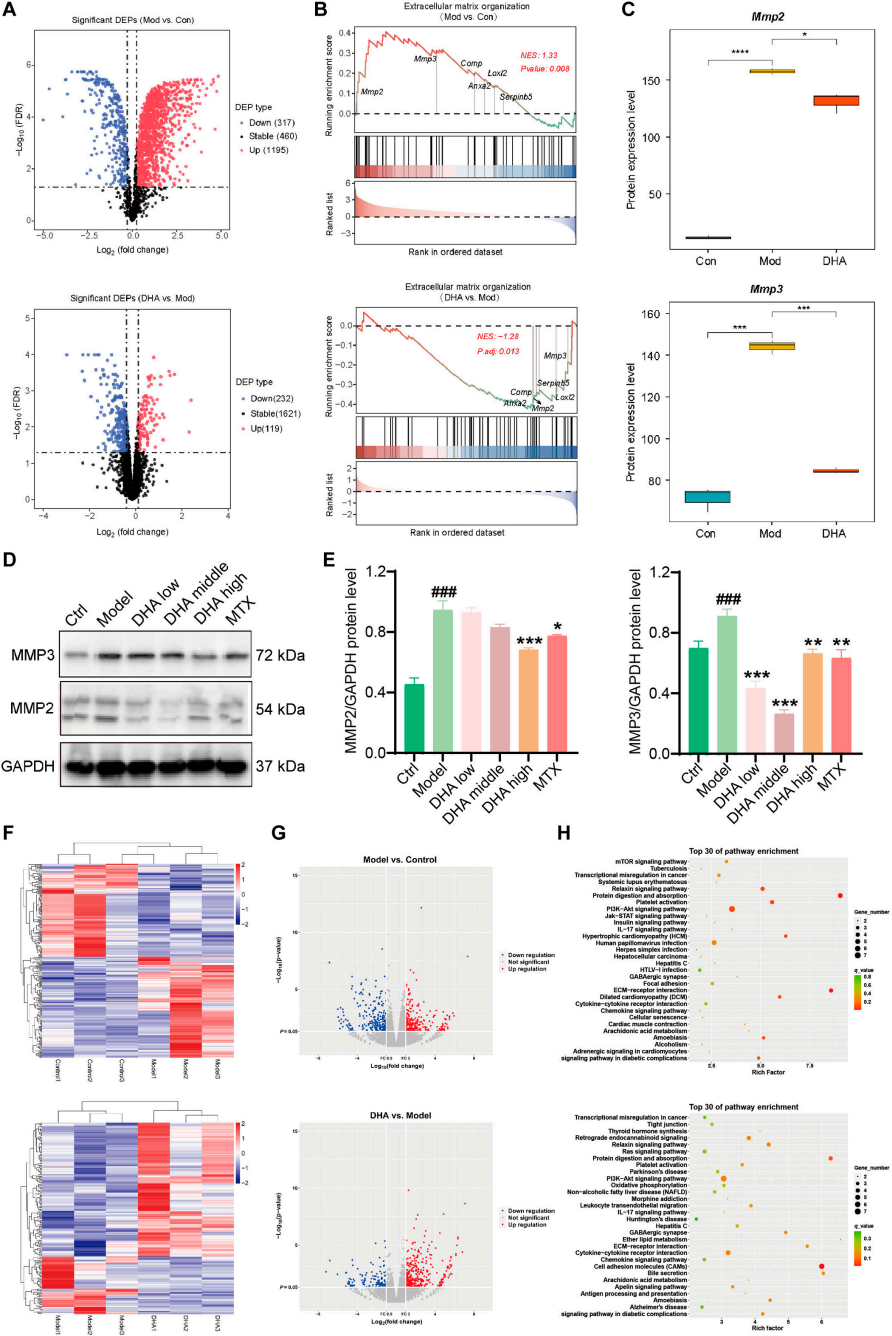
Effect of DHA on hippocampus and synovial protein expression in RA rats. (A) The volcano plots display the expressions of differentially expressed proteins (DEPs) in both MVC and MVD groups. (B) The rank in the ordered dataset reveals DEPs involved in the extracellular matrix (ECM) organization signaling pathway. (C) Proteomics datasets indicate the expression levels of MMP2 and MMP3. (D) WB validation was conducted on MMP2 and MMP3. (E) Statistical analysis of WB. The data are presented as the mean ± SD (*n* = 3). ^###^*P* < 0.001, significance between Model versus Ctrl; *P < 0.05, **P < 0.01, ***P < 0.001, significance between DHA or MTX versus Model. (F) The heatmap plots illustrate the relative expressions of DEGs in MVC and MVD groups. (G) The volcano plots depict the relative expressions of DEGs in MVC and MVD groups. (H) The KEGG analysis reveals the enriched pathways in MVC and MVD groups.

The single-cell transcriptome analysis results revealed that the alterations of fibroblasts, macrophages, and B cells in the synovial tissue of DHA-treated RA rats were all associated with the ECM at the genetic level. Analysis of the types, amounts, and functions of proteins in rat synovial tissues by proteome sequencing, combined with the results of scRNA-seq, can reveal the biological processes of DHA anti-RA in rats. In light of this, whole-proteome sequencing was performed on knee synovial tissues obtained from both RA rats and DHA-treated rats. By focusing on the ECM signaling pathway, it was observed that DHA could modulate the cellular microenvironment by down-regulating the protein expression levels of MMP2 and MMP3 (Fig. 5A to C). Under RA conditions, inflammatory factors can stimulate the production of MMPs, which further lead to irreversible cartilage and bone damage. According to our validation experiments, both MMP2 and MMP3 showed an increasing trend in RA rats, which was reversed by DHA administration (Fig. 5D and E).

To elucidate the mechanism of DHA in alleviating pain symptoms of RA rats, transcriptome sequencing was conducted on the hippocampus. Interestingly, the DEGs between the DHA group and the Model group were also found to be enriched in the ECM receptor interaction pathway (Fig. 5F to H). This provides further evidence that DHA acts on the ECM and exerts a systemic, top-down effect from the central to peripheral regions in RA rats.

### DHA effectively reduces mitochondrial membrane potential and promotes apoptosis of MH7A cells

**Fig. 6.**
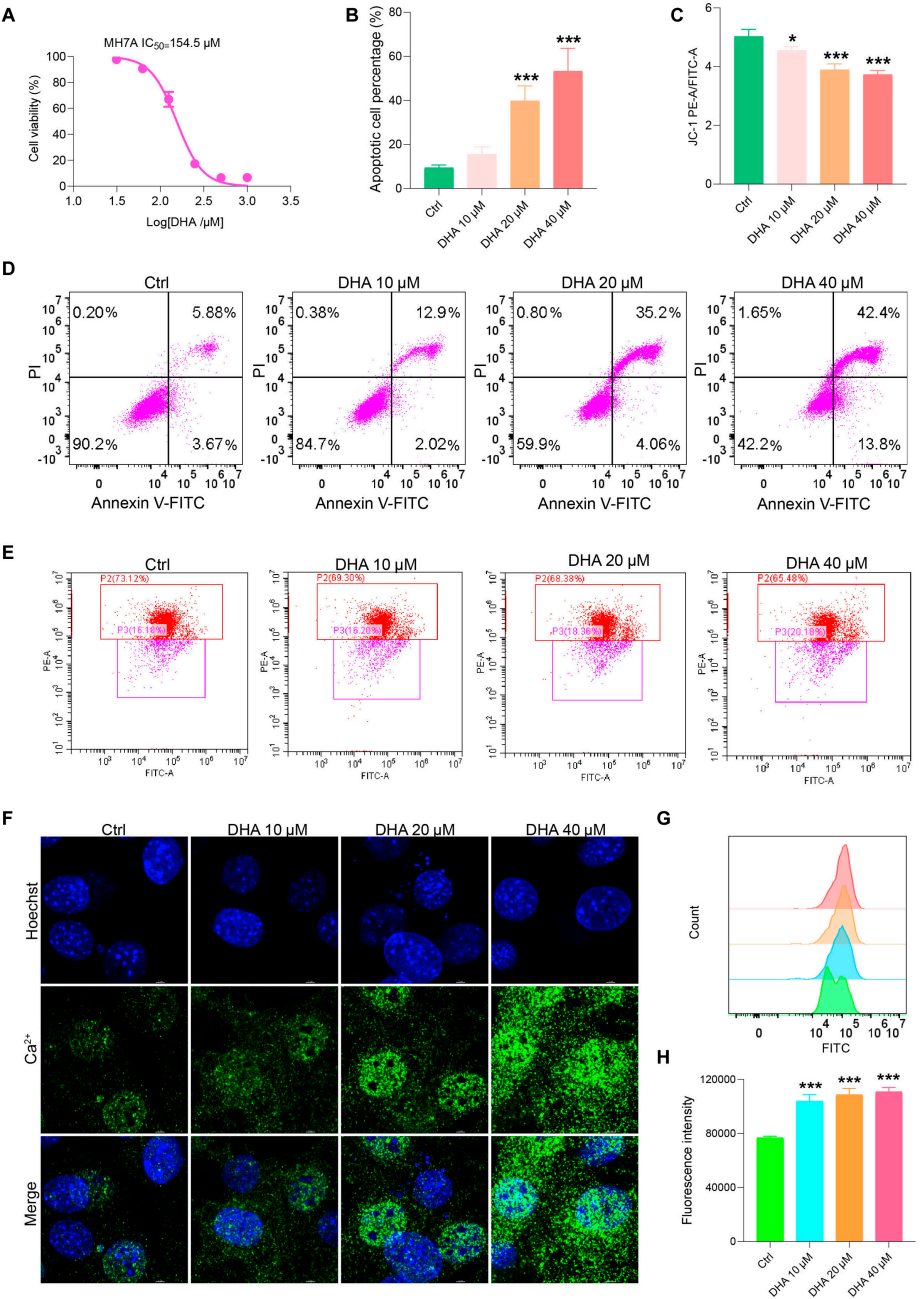
Effects of DHA on mitochondrial membrane potential, apoptosis, and the intracellular Ca^2+^ concentration of MH7A cells. (A) Impact of DHA on the cell viability of MH7A cells (*n* = 3). (B) The cell apoptosis rate was quantified. (C) The ratio of red–green fluorescence intensity indicating mitochondrial membrane potential was quantified. (D) MH7A cells were analyzed for apoptosis (*n* = 3). (E) MH7A cells were analyzed for mitochondrial membrane (*n* = 3). (F) Intracellular Ca^2+^ concentration in MH7A cells was detected using fluorescence imaging. (G) Flow cytometry was performed to quantify the intracellular Ca^2+^ concentration in MH7A cells. (H) Statistical analysis of Ca^2+^ concentration. The data are presented as the mean ± SD. *P < 0.05, **P < 0.01, ***P < 0.001, compared to the Ctrl group.

The abnormal proliferation of synovial fibroblasts under RA condition contribute to both synovitis and joint damage [25]. Thus, inducing apoptosis of synovial fibroblasts is one of the therapeutic strategies against RA. Apoptosis is the autonomous and orderly death of cells controlled by genes in order to maintain the stability of the internal environment, and the decrease in mitochondrial membrane potential is a hallmark event in the early stage of apoptosis. To investigate the influence of DHA on synovial fibroblasts, we detected the cell apoptosis as well as mitochondrial membrane potential on the basis of MH7A synovial fibroblasts. We found that the survival rate of MH7A cells decreased to 50% when DHA was used at a working concentration of approximately 154.5 μM (Fig. 6A). Flow cytometry was employed to measure cell apoptosis and mitochondrial membrane potential. The apoptosis rate of MH7A cells significantly increased following DHA intervention, with a notable rise in the proportion of late apoptotic cells in the 20 μM and 40 μM groups (Fig. 6B). In the early phase of apoptosis, the mitochondrial membrane potential decreased, as evidenced by a reduced red/green fluorescence ratio in MH7A cells across all 3 DHA concentrations (Fig. 6C). Flow scatterplots provided visual representations of cellular changes (Fig. 6D and E). The confocal microscopy fluorescence imaging revealed that the administration of DHA at concentrations of 10, 20, and 40 μM resulted in enhanced Ca^2+^ concentrations in MH7A cells (Fig. 6F). Flow cytometry was employed for further quantification, demonstrating that all 3 concentrations of DHA significantly increased the fluorescence intensity of Ca^2+^ in MH7A cells in contrast with the untreated cells (*P* < 0.05; Fig. 6G and H).

## Discussion

Although DHA has gained increasing attention in the application of RA control, its underlying mechanisms are still not fully understood, which hampers its widespread application. The AIA model is a classic animal model used to study the efficacy and mechanisms of RA [26,27]. In our study, we observed that DHA effectively reduced arthritis scores and joint swelling, and effectively inhibited the secretion of cytokines IL-17, MMP9, TRAP, and CTSK. Additionally, DHA improved the reduction in mechanical pain threshold, indicating that it could effectively reduce inflammatory pain phenotype of arthritis, which is closely related to hippocampal tissue. Based on analysis of hippocampal tissue samples of rats in different groups, we found that DHA was regulated on the ECM by regulating of the central region of RA rats. Furthermore, DHA notably prevented bone loss in the ankle as well as the knee joints of the model rats. Moreover, in our in vitro experiments using the MH7A synovial fibroblast cell line, we found that DHA promoted synovial fibroblast cell apoptosis and markedly increased the fluorescence intensity of intracellular calcium (Ca^2+^) in MH7A cells. Collectively, our study elucidated the anti-RA effect of DHA both in vivo and in vitro.

In recent years, there have been several reports applying scRNA-seq to the study of RA diseases and finding that synovial ECM is crucial in the pathogenesis of RA [28]. DHA has been found to dramatically modulate the structural components of the ECM [16], but has not yet been studied at the single-cell level. To systematically investigate the mechanism of action of DHA, single-cell sequencing technology was used to construct the first single-cell profile of the knee synovium joint of DHA-treated RA rats and further analyzed the gene expressions in several major cell types to elucidate the molecular and cellular events involved in DHA treatment of RA. Through the single-cell profiling, we identified a total of 8 cell types in the synovial tissues according to marker genes: fibroblasts, endothelial cells, myocytes, B cells, macrophages, neutrophils, platelets, and red blood cells. DHA intervention induced major changes in 4 types of cells, namely, fibroblasts, macrophages, B cells, and endothelial cells. The synovium of the joint consists of sublining and lining layers, with the lining layer composed of fibroblasts and macrophages, along with the vascular network, forming the sublining layer [29]. Synovial fibroblasts, together with macrophages, contribute to joint destruction by producing pro-inflammatory cytokines, chemokines, and components of ECM remodeling [30–33].

Synovial fibroblasts, as significant immune players in the synovial membrane, interact with macrophages and other immune cells to secrete pro-inflammatory cytokines and tissue-degrading factors including IL-6, IL-8, and MMPs [34–36], which further perpetuate inflammation and bone erosion [37,38]. In our study, RNA-seq analysis was employed to examine the genetic and functional changes in RA synovial fibroblast subsets treated with DHA. GO enrichment analysis of DEGs suggested that the up-regulated genes in the Model group were mainly associated with functional pathways related to ECM composition and response to TGF-β. The down-regulated genes after DHA treatment also exhibited enrichment in ECM composition. These findings confirmed that DHA can modify the microenvironment of synovial tissue in RA rats.

The aggravation process of RA involves various immune cells, including adaptive immune macrophages and innate immune B cells [39–41]. Macrophages exhibit 2 phenotypes: pro-inflammatory (M1) and anti-inflammatory (M2). Notably, the anti-RA drug MTX has been designed for targeted delivery into M1 macrophages [42,43]. In addition to the direct effects of MTX on M1 macrophages, interventions can be implemented to convert M1 macrophages into M2 macrophages by modifying the microenvironment at the site of joint lesions [44,45]. Our findings indicate that DHA treatment significantly reduced the ratio of M1 macrophages to M2 macrophages in RA rats, with DEGs primarily associated with the ECM structural component signaling pathway. These results suggest that DHA inhibits the M1 phenotype while promoting M2 macrophage polarization through the extracellular microenvironment, which aligns with previous studies. In summary, DHA shows potential as a treatment option for RA by modulating the ratio of M1/M2 macrophages.

B cells play an intermediate role between innate and adaptive immunity. In RA, chemokines secreted by fibroblasts adhere to the cell surface, leading to the recruitment of B cells and promoting inflammation and cartilage damage [46]. Additionally, they contribute to B cell differentiation and activation [47]. The signaling between fibroblasts and B cells is bidirectional, as B cells can stimulate synovial fibroblasts to secrete more inflammatory cytokines such as IL-6, thereby triggering the inflammatory process [48]. In the Model group, the total number of 3 B cell subtypes increased and was restored by DHA treatment. In most B_C1 subtypes, pathways associated with ECM structural components were down-regulated after DHA treatment compared to the Model group. These findings indicate that DHA effectively regulate the extracellular mechanisms of synovial B cells in RA rats. Therefore, DHA may inhibit B cell activation by influencing the B cell immune microenvironment.

The acute development of RA is accompanied by a sustained immune and inflammatory response, which induces endothelial cell activation, damage, and dysfunction. Endothelial cells maintain joint stability by facilitating the exchange of soluble factors between synovial fluid and blood circulation [49]. In RA, synovial fibroblasts migrate to distal cartilage through the vascular system. Excessive angiogenic factors counteract angiogenesis inhibitors, support increased endothelial cell infiltration, promote synovial inflammation, and destroy bone and cartilage [50]. Drugs such as PDE5 inhibitors ameliorate RA-induced walking impairment and pain by targeting endothelial function [51]. In addition, MTX has been shown to play a role in inhibiting angiogenesis [52]. The results of single-cell transcriptome analysis and immunofluorescence images were consistent and showed that the expression levels of both endothelial cell markers, VEGFC and CD31, were significantly reduced in rat synovial tissues after DHA administration. These results suggest that DHA alleviates RA by inhibiting endothelial cell angiogenesis.

The single-cell transcriptome analysis conducted in this study revealed that the alterations observed in fibroblasts, macrophages, and B cells in the synovial tissue of DHA-treated rats were all related to the ECM at the genetic level. Previous research has demonstrated that RA induces inflammatory invasion at synovial sites, leading to ECM degradation through increased activity of MMPs, ultimately resulting in irreversible cartilage damage. This highlights the significance of MMPs in joint injury [53]. In light of this, whole-proteome sequencing was performed on knee synovial tissues obtained from both RA rats and DHA-treated rats. By focusing on the ECM signaling pathway, it was observed that DHA could modulate the cellular microenvironment by down-regulating the protein expression levels of MMP2 and MMP3.

To investigate the central pathway through which DHA influences pain representation in RA rats, transcriptome sequencing was conducted on rat hippocampus. Interestingly, the DEGs between the DHA-treated group and the Model group were found to be enriched in the ECM receptor interaction pathway. This provides further evidence that DHA indeed acts on the ECM and exerts a systemic, top-down effect from the central to peripheral regions in RA rats.

To validate the findings from the bioinformatics analysis, Western blot (WB) experiments were performed on the synovial tissue of rats to measure the levels of MMP2 and MMP3. However, it should be noted that the effects of DHA on the ECM are not solely confined to MMPs, and further investigation is required to explore other mechanisms. In conclusion, this study elucidated that DHA regulates the cellular microenvironment mediated by MMPs to alleviate RA, employing a combination of RNA-Seq, proteomics, transcriptomics, and experimental validation. Nevertheless, while MMPs represent important targets in the regulation of DHA on RA, other potential mechanisms warrant further research in the future. In general, in this study, we discover that DHA has potential to be used in the treatment of RA by inhibiting inflammation response, reducing pain, and relieving joint injuries by regulating the synovial tissue microenvironment as well as the hippocampal tissue. However, the direct targeted proteins of DHA and their mediated pathways on the major cell types still needed further investigation. So, activity-based protein profiling and cellular thermal shift assay, which provide drug probe-protein profile, will be applied for further studies in the future [54,55]. In addition, due to the short half-live, the nanoparticle drug delivery systems of DHA, which actively target inflamed joints, will help to improve its therapeutic efficacy in the treatment of RA [56].

## Materials and Methods

### Animals

Male Lewis rats aged 8 to 10 weeks were obtained from Beijing Vital River Laboratory Animal Technology Co. Ltd. (Beijing, China), the animal license number of which is SYXK (Beijing, China) 2021-0011. The rats were housed in the Animal Room of the Institute of Chinese Medicine, China Academy of Chinese Medical Sciences. The animal research protocol has been approved by the Animal Ethics Committee (protocol code: 2022B116).

### Induction of AIA

Fifty male Lewis rats were randomly and evenly assigned to 5 groups: Control, AIA model, AIA model treated with low-dose DHA (15 mg/kg), AIA model treated with high-dose DHA (30 mg/kg), and AIA model treated with MTX (0.2 mg/kg) groups (*n* = 10 in each group). The AIA modeling method followed the procedure described in a previous study [57]. Briefly, rats in the Model and treatment groups received a single intravenous injection in the tail of 10 mg/ml M tuberculosis H37 Ra (Becton, Dickinson and Company, Sparks, MD 21152, USA) dissolved in liquid paraffin. The normal rats in the Control group received an equivalent volume of saline instead. Subsequently, the rats in the treatment groups were orally administered DHA once a day or MTX twice a week for a consecutive period of 4 weeks. The Control and Model groups underwent the same procedure but received an equal volume of distilled water. DHA with a purity of at least 98.9% was purchased from MedChemExpress.

### Mechanical pain was measured with von Frey pain measuring filament

The mechanical pain threshold in the L5 reflex area of the left foot in rats was assessed following the methodology described in our previous study [58]. In brief, the rats were placed in a metal cage, ensuring that they were quiet and awake during the experiment. The paw surface of the rats was vertically stimulated using von Frey filaments with varying forces. A positive response was recorded when there was a slight bending of the filaments for 5 s or until the paw withdrawal reaction occurred. The 50% paw withdrawal response threshold was calculated using the up and down method. Three cycles of measurements were performed, and the mean value was obtained for each rat.

### Clinical assessment of AIA

The rats were closely monitored by examiners daily, and the arthritis score was recorded every other day. Additionally, joint swelling and ankle diameter were assessed as general indicators at the conclusion of the drug administration period.

### Micro-CT

After anesthesia, the right ankle and knee joints were dissected and fixed in 4% paraformaldehyde for 72 h. The joints were then scanned using a micro-CT system (Skyscan 1174, Bruker micro-CT, Kontich, Belgium) with the following parameters: resolution of 25 μm and an exposure time of 72 min. Analysis of bone volume, bone volume percentage, and bone surface/volume ratio was performed using CTAn V 1.13 software (Bruker micro-CT), an image analysis system.

### Cytokine assays

Blood samples were collected from the rat’s abdominal aorta and left at room temperature for 1 h. After centrifugation at 3,500 rpm for 10 min, the supernatant was collected for the detection of IL-17, MMP9, TRAP, and CTSK cytokines using enzyme-linked immunosorbent assay (ELISA) kits (Jiangsu Jingmei Biotechnology company with limited liability, Jiangsu, China).

### Hematoxylin and eosin staining

The knee joints of the rats, which had been fixed with 4% paraformaldehyde, were embedded in paraffin and sectioned. The paraffin sections were then dewaxed to water, stained with hematoxylin and eosin (H&E), dehydrated, and sealed. The results were observed using a microscope.

### Single-cell isolation, library construction, and data preprocessing

The synovial tissues of the rats’ knee joints were collected from the Control, Model, and DHA (30 mg/kg) groups using the rat synovial dissociation kit according to the provided instructions. Briefly, the samples were filtered through a 70-mm filter, rinsed with Dulbecco’s modified Eagle’s medium (DMEM), and centrifuged at 300*g* for 10 min to collect the cells. The collected cells were then resuspended in 1 ml of phosphate-buffered saline (PBS), and the red blood cells were removed. The resulting single-cell suspension was prepared by washing twice with PBS and subsequently diluted to a concentration of 1,000 cells/μl. Droplets were captured, nanoscale gel beads were generated, and cDNA was isolated and purified through reverse transcription for polymerase chain reaction (PCR) amplification. The amplified cDNA was used to construct an RNA-seq library, which underwent further purification, quality evaluation, and sequencing on the Illumina HiseqXTEN platform. Low-quality cells were filtered out, and the high-quality cell data were integrated for cell clustering. To visualize and analyze all the cells, the Uniform Manifold Approximation and Projection (UMAP) algorithms were utilized.

### Cell classification and DEG analysis

Seurat’s FindAllMarkers function was employed to classify individual cells based on genes known to be specifically expressed in distinct cell types and subpopulations. Principal components analysis (PCA), clustering, and annotation techniques were then used to identify cell subtypes. Violin plots or heatmaps were generated using the Seurat VlnPlot function and the R packages pheatmap (v1.0.12), adhering to the criteria of |avg_log2FC| > 0.25, pct > 0.1, and adjusted *P* < 0.05. Furthermore, GO analysis was conducted using the ClusterProfiler R package (version 3.18.1) to explore the intersection of DEGs among various groups and to identify GO enrichment.

### Proteomics and data analysis

To perform whole-proteome sequencing, dithiothreitol (DTT) and iodoacetamide (IAA) were added to the synovial tissues obtained from both RA rats and DHA-treated (30 mg/kg) rats groups. The mixture was then subjected to cold acetone and kept at −80 °C for 30 min. After centrifugation, the excess acetone was dissolved, and the remaining components were precipitated using 100 mM tetraethylammonium bromide (TEAB). The precipitated sample was subsequently digested with trypsin at 37 °C overnight. Following another round of centrifugation, the supernatant was passed through a C18 column at a reduced speed. The sample was desalted using 0.1% formic acid and then eluted with a 0.1% formic acid–50% acetonitrile solution. The eluent was then dried using a centrifugal process. The DEG samples, identified based on an absolute fold change of ≥2 and an adjusted *P* value (false discovery rate) of <0.05, were subjected to analysis using liquid chromatography–tandem mass spectrometry (Thermo Orbitrap Fusion Lumos, USA). Furthermore, the data obtained from this analysis were visualized using GO analysis and volcano maps.

### Transcriptomics and data analysis

RNA was extracted from the hippocampal tissues of rats belonging to the Control, Model, and DHA (30 mg/kg) groups (*n* = 3) for transcriptome sequencing. The objective was to investigate the underlying mechanisms behind the analgesic effect of DHA. The samples were prepared following the instructions provided by TruSeq RNA, and subsequently, cDNA libraries were generated through PCR amplification and purification. The libraries were then sequenced using the Illumina NovaSeq 6000 platform (Illumina, USA). Fastq data files were processed using a Perl script, and the DEGs were identified based on the criteria of |log_2_ fold change| ≥ 1 and *P* < 0.05. These DEGs were further subjected to enrichment analysis and analyzed using the Kyoto Encyclopedia of Genes and Genomes (KEGG) database.

### Cell culture and treatment

MH7A cells were cultured in a 37 °C incubator with a 5% CO_2_ concentration, using DMEM supplemented with 10% fetal serum and 1% penicillin. Once the MH7A cells reached 80% to 90% confluence, 5,000 cells were seeded in each well of 96-well plates. The cells were then exposed to varying concentrations of DHA (0, 1, 5, 10, 20, 40, 60, 80, and 100 μM) for a duration of 24 h. After the exposure period, 10 μl of CCK-8 solution (obtained from Keygen Biotech Co. Ltd., Nanjing) was added to each well and incubated at 37 °C for 2 h. The optical density (OD) value of each well was measured using an enzyme marker, and subsequently, the median inhibitory concentration (IC_50_) value was calculated based on the obtained data.

### Detection of apoptosis, mitochondrial membrane potential, and Ca^2+^ concentration

MH7A cells were treated with different concentrations of DHA, specifically 20, 40, and 80 μM, for a duration of 24 h. To detect apoptosis, annexin V–fluorescein isothiocyanate (FITC)/propidium iodide (PI) double staining was employed. The mitochondrial membrane potential was assessed using the JC-1 dye, and the Ca^2+^ concentration was labeled using the fluo-4 AM probe. All procedures were conducted following the instructions provided in the Beyotime Biotechnology Kit. Flow cytometry analysis was performed using a flow cytometer (Beckman, based in Indiana, USA).

### WB experimental verification

The synovial tissues of rats were extracted using radioimmunoprecipitation assay (RIPA) cracking solution to extract proteins. The proteins, with varying molecular weights, were then separated using sodium dodecyl sulfate–polyacrylamide gel electrophoresis (SDS-PAGE) gel at a voltage of 120 V. Subsequently, the proteins were transferred onto a polyvinylidene difluoride (PVDF) membrane through electric transfer. The membrane was blocked using milk and then incubated overnight at 4 °C with the primary antibody. On the following day, the membrane was washed, and the corresponding secondary antibody was added and incubated at room temperature with shaking for 2 h. The protein bands were visualized using an enzyme-linked chemiluminescence system (Azure C400 system, USA). The intensity of the protein bands was quantified using image analysis software such as ImageJ. The primary antibodies used, including anti-MMP2 and anti-MMP3, were obtained from Proteintech Abcam (Cambridge, UK).

### Statistical analysis

Statistical analysis was performed using GraphPad Prism software (version 8.0, San Diego, CA, USA). One-way repeated-measures analysis of variance (ANOVA) was used to analyze the differences between groups. The results were presented as mean ± standard deviation (SD). A significance level of *P* < 0.05 was considered statistically significant.

## Data Availability

The data of this paper have been deposited in the OMIX, China National Center for Bioinformation/Beijing Institute of Genomics, Chinese Academy of Sciences (https://ngdc.cncb.ac.cn/omix: accession no. OMIX005405).
