# Supplementary material for: Dihydroartemisinin Regulated the MMP-Mediated Cellular Microenvironment to Alleviate Rheumatoid Arthritis
Source: Research (Wash D C). 2024 Sep 10;7:0459. doi: 10.34133/research.0459 (PMC11385568; doi:10.34133/research.0459)
Supplement: Supplementary 1 — Fig. S1 [file research.0459.f1.zip › Supplementary Material.docx]

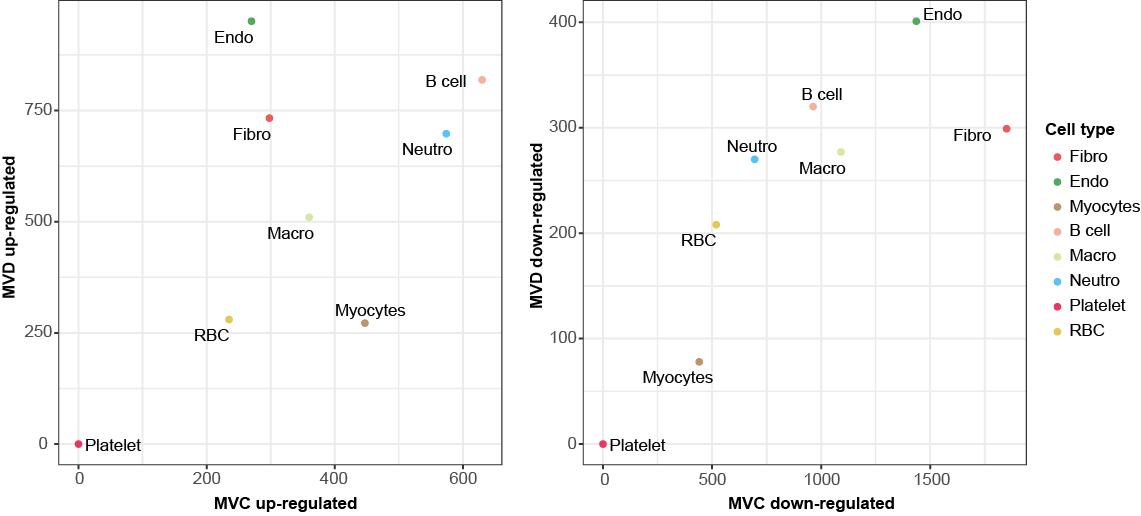


**Fig. S1.** Differential expressed genes (DEGs) of Model vs. Ctrl (MVC) and Model vs. DHA (MVD) groups, respectively. The dot plot indicates the numbers of differential expressed genes (DEGs) from Model vs. Ctrl (MVC, X axis) and Model vs. DHA (MVD, Y axis) groups, dividing into the up-regulated (left panel) and down-regulated (right panel) patterns, colored by cell types.
