# Supplementary figures and images for: Dihydroartemisinin Regulated the MMP-Mediated Cellular Microenvironment to Alleviate Rheumatoid Arthritis
Source: Research (Wash D C). 2024 Sep 10;7:0459. doi: 10.34133/research.0459 (PMC11385568; doi:10.34133/research.0459)

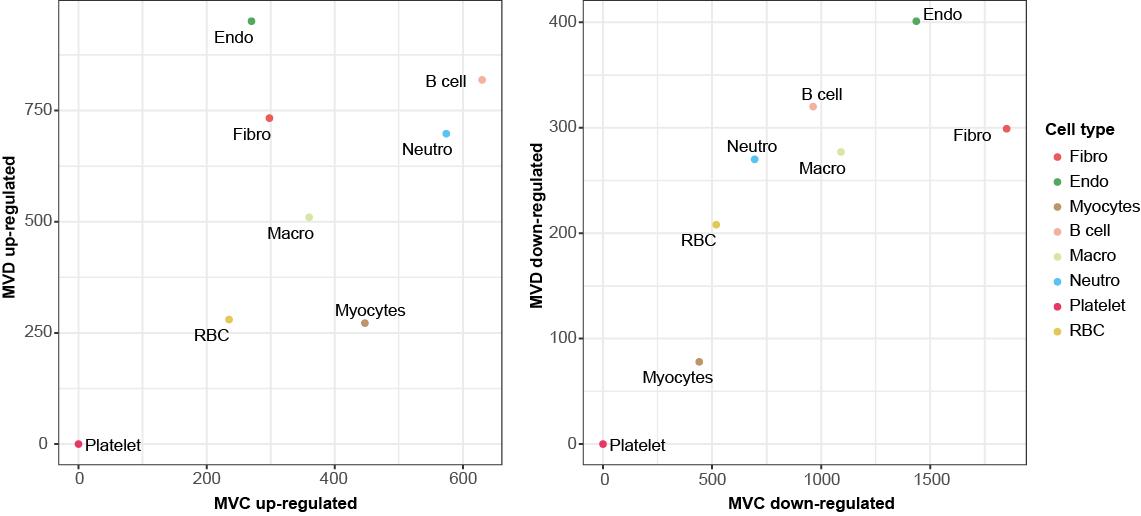

Supplement: Supplementary 1 — Fig. S1 [file research.0459.f1.zip › Figure S1.jpg]
